# Tracing regulatory routes in metabolism using generalised supply-demand analysis

**DOI:** 10.1186/s12918-015-0236-1

**Published:** 2015-12-03

**Authors:** Carl D. Christensen, Jan-Hendrik S. Hofmeyr, Johann M. Rohwer

**Affiliations:** Laboratory for Molecular Systems Biology, Department of Biochemistry, Stellenbosch University, Private Bag X1, Matieland, Stellenbosch, 7602 South Africa; Centre for Studies in Complexity, Stellenbosch University, Private Bag X1, Matieland, Stellenbosch, 7602 South Africa

**Keywords:** Generalised supply-demand analysis, Metabolic control analysis, Metabolic modelling, *Arabidopsis thaliana*, *Lactococcus lactis*

## Abstract

**Background:**

Generalised supply-demand analysis is a conceptual framework that views metabolism as a molecular economy. Metabolic pathways are partitioned into so-called supply and demand blocks that produce and consume a particular intermediate metabolite. By studying the response of these reaction blocks to perturbations in the concentration of the linking metabolite, different regulatory routes of interaction between the metabolite and its supply and demand blocks can be identified and their contribution quantified. These responses are mediated not only through direct substrate/product interactions, but also through allosteric effects. Here we subject previously published kinetic models of pyruvate metabolism in *Lactococcus lactis* and aspartate-derived amino acid synthesis in *Arabidopsis thaliana* to generalised supply-demand analysis.

**Results:**

Multiple routes of regulation are brought about by different mechanisms in each model, leading to behavioural and regulatory patterns that are generally difficult to predict from simple inspection of the reaction networks depicting the models. In the pyruvate model the moiety-conserved cycles of ATP/ADP and NADH/NAD ^+^ allow otherwise independent metabolic branches to communicate. This causes the flux of one ATP-producing reaction block to increase in response to an increasing ATP/ADP ratio, while an NADH-consuming block flux decreases in response to an increasing NADH/NAD ^+^ ratio for certain ratio value ranges.

In the aspartate model, aspartate semialdehyde can inhibit its supply block directly or by increasing the concentration of two amino acids (Lys and Thr) that occur as intermediates in demand blocks and act as allosteric inhibitors of isoenzymes in the supply block. These different routes of interaction from aspartate semialdehyde are each seen to contribute differently to the regulation of the aspartate semialdehyde supply block.

**Conclusions:**

Indirect routes of regulation between a metabolic intermediate and a reaction block that either produces or consumes this intermediate can play a much larger regulatory role than routes mediated through direct interactions. These indirect routes of regulation can also result in counter-intuitive metabolic behaviour. Performing generalised supply-demand analysis on two previously published models demonstrated the utility of this method as an entry point in the analysis of metabolic behaviour and the potential for obtaining novel results from previously analysed models by using new approaches.

**Electronic supplementary material:**

The online version of this article (doi:10.1186/s12918-015-0236-1) contains supplementary material, which is available to authorized users.

## Background

The primary concern of molecular biology is to identify and characterise the individual components of biological systems. By focussing on the component level, this approach has generated an enormous amount of knowledge, but at the expense of disregarding emergent phenomena that arise from the multitude of interactions between these components. One way of overcoming this limitation is to construct, and subsequently study, mathematical models of these biological systems. This technique has become increasingly common, with models describing systems ranging in complexity from metabolic subsystems to genome-scale reconstructions of metabolism [1, 2] being available on various online databases [3–5]. More recently the goal of building a silicon cell [6] was arguably realised with the construction of a whole-organism model of *Mycoplasma genitalium* [7]. With models growing in size and complexity, approaching that of the modelled systems themselves, it has become more difficult to extract biological knowledge and understanding from them without extensive analysis. Model construction is therefore only the first step in the study of biological systems using the ‘modelling approach’.

Generalised supply-demand analysis (GSDA) is a conceptual framework that views metabolic pathways as a molecular economy [8]. It is built on the principles of metabolic control analysis (MCA) [9, 10], which is itself a framework that allows for the quantification of the control that any step in the system exercises over the variable steady-state properties such as fluxes or intermediate metabolite concentrations. The basic procedure of supply-demand analysis is to divide a metabolic pathway into separate reaction blocks around a chosen variable metabolite by fixing its concentration; the ‘generalised’ in GSDA implies that this is done in turn for each metabolite in the system. The supply and demand blocks, which respectively produce and consume the fixed metabolite, are subsequently treated as separate metabolic units, and MCA is performed on each reaction block. This approach allows for the identification of certain regulatory features of the pathway and for the quantification of the behaviour of the reaction blocks towards to changes in the concentration of the fixed metabolite. One such feature that GSDA helps to identify and quantify is the effect of different routes of interaction between the variable metabolites and their supply and demand reaction blocks.

The simplest way that reaction blocks can interact is through the common intermediate that links them, which can serve as either substrate or product or allosteric effector of supply and demand enzymes. If the only interactions are via the linking metabolite the situation is easy to analyse. However, it is possible that reaction blocks also interact indirectly through allosteric effects of a metabolite in one reaction block on an enzyme in the other reaction block; such a situation is quite common and it is here that GSDA is particularly useful in that it dissects all the routes of communication between supply and demand, both direct and indirect.

Another common situation is where cofactor cycles such as ATP–ADP or NADH–NAD ^+^ link supply and demand reaction blocks. These cycles are called moiety-conserved cycles because they interconvert different forms of a chemical subgroup or moiety, the total concentration of which remains constant. For example, in the ATP/ADP cycle the moiety is ADP, which is interconverted between its free and its phosphorylated form. When there is no *de novo* synthesis or degradation of the ADP-moiety during the time-scale of observation, the sum of ADP and ATP remains constant even while their individual concentrations change, and the cycle is therefore moiety-conserving. In supply-demand analyses of such cycles the relevant variable that links the supply and demand reaction blocks is not a single concentration but rather a concentration ratio such as ATP/ADP or NADH/NAD ^+^. These cycles usually form metabolic hubs where many functionally distinct pathways are integrated; an analysis of the interplay between supply and demand around these cycles is crucial for our understanding of metabolic regulation.

In this paper we use GSDA to investigate the regulatory effects brought about by multiple routes of interaction between supply and demand reaction blocks. We have chosen to analyse models of two metabolic pathways that differ from each other in terms of their regulatory mechanisms. The first is a model of pyruvate metabolism in lactic acid bacteria [11] where multiple interactions are brought about through the moiety-conserved cycles of ATP/ADP and NADH/NAD ^+^. We show that changes in the ratios of the different forms of a moiety can cause counter-intuitive responses in reaction block fluxes. The second is a model of aspartate-derived amino acid synthesis in *Arabidopsis thaliana* [12] in which we analyse the routes of interaction brought about by allosteric effectors in combination with multiple isoenzymes. Here we explore the functions of the various isoenzymes and construct a map that shows the importance of the routes of regulation between a fixed metabolite and its supply block. In both models the importance of each route of interaction originating from a change in the fixed metabolite will be quantified and compared, illustrating that the direct route of interaction is not necessarily the most important. More generally we demonstrate the utility of investigating previously published models with a new analytic tools such as GSDA.

## Methods

### Metabolic control analysis

Metabolic control analysis (MCA) is a form of sensitivity analysis in which the control properties of a steady-state metabolic system are quantified in terms of the responses of the system fluxes and metabolite concentrations to perturbations in the rates of the reactions [9, 10]. Because this framework plays a central role in generalised supply-demand analysis [8], we here define its three main coefficients and their relation to each other.

An *elasticity coefficient* describes the sensitivity of a reaction rate towards a change in any entity *x* that can affect that rate directly, such as a substrate, product, modifier or enzyme parameter. It is therefore a property local to a particular reaction and is defined as the ratio of the relative change in the rate of reaction *i*, *v*_*i*_, to the relative change in *x*:

(1) $$ \varepsilon^{v_{i}}_{x}=\frac{\partial \ln\, v_{i}}{\partial \ln\, x}  $$

A *control coefficient* describes the sensitivity of a steady-state system variable, such as flux or concentration, towards a change in a local reaction rate. This coefficient is a systemic property that depends not only on the properties of the perturbed reaction but also on those of the other reactions and the topology of the network structure of the entire pathway. The flux-control coefficient is defined as the ratio of the relative change in a flux, *J*, to the relative change in a reaction rate, *v*_*i*_:

(2) $$ C^{J}_{v_{i}}=\frac{d \ln\, J}{d \ln\, v_{i}}  $$

Concentration-control coefficients are defined similarly, the flux being replaced by a metabolite concentration. The use of a total derivative signifies that the entire system is allowed to relax to a new steady state after the perturbation in *v*_*i*_.

A *response coefficient* differs from a control coefficient in that it is defined with respect to a change not in a local reaction rate but in a system parameter, such as enzyme concentration or the fixed concentration of metabolite external to the system. A flux-response coefficient is defined similarly to a control coefficient as the ratio of the relative change in a flux, *J*, to the relative change in parameter *x*:

(3) $$ {R^{J}_{x}} = \frac{d \ln\, J}{d \ln\, x}  $$

Again, in a concentration-response coefficient metabolite concentration replaces flux.

The so-called partitioned response (or combined-response) equation describes the relationship between these three coefficients:

(4) $$ {R^{J}_{x}} = C^{J}_{v_{i}} \varepsilon^{v_{i}}_{x}  $$

The overall flux-response to a perturbation in parameter *x* is channelled through the reaction *i* directly affected by *x*: the effect of *δx* on *v*_*i*_ is described by $\varepsilon ^{v_{i}}_{x}$, and the resulting change *δv*_*i*_ then propagates through the system resulting in a change in flux described by $C^{J}_{v_{i}}$. If the parameter *x* affects more than one reaction, the overall flux-response is given by

(5) $$ {R^{J}_{x}} = \sum_{i} C^{J}_{v_{i}} \varepsilon^{v_{i}}_{x}   $$

for all reactions *i* that are influenced directly by *x*.

### Generalised supply-demand analysis

**Fig. 1:**
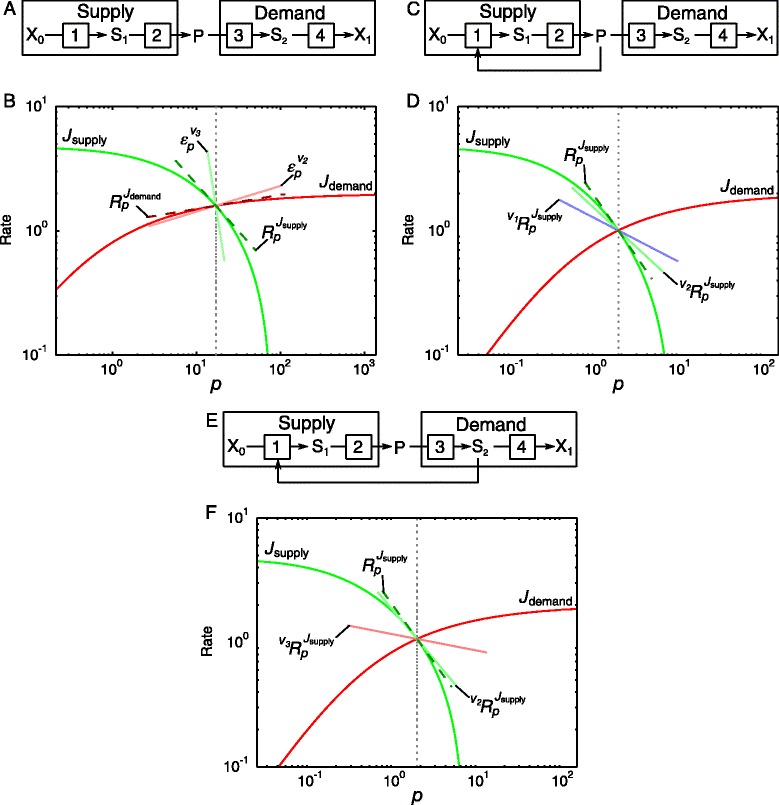
An example of generalised supply-demand analysis of three metabolic systems. **a** An example of a simple linear pathway partitioned into supply and demand blocks around intermediate P. **b** A rate characteristic plot that shows how the fluxes local to the supply and demand blocks of P respond to a change in *p* over a large concentration range. The vertical dotted line indicates the steady-state concentration of P ($\bar {p}$), while the steady-state flux of the system as a whole obtains where the rates of the supply and demand blocks intersect. Elasticity coefficients of the reactions in the supply and demand blocks that interact directly with P (reactions 2 and 3 respectively) are indicated with solid lines while response coefficients of the blocks towards *p* are indicated by dashed lines. **c** The pathway in (**a**) with the addition of allosteric inhibition of enzyme 1 by P, which creates an additional direct route of interaction between P and its supply block via reaction 1. **d** The rate characteristic plot for the supply and demand blocks of the intermediate P in (**c**). **e** The pathway in (**a**) with the addition of allosteric inhibition of enzyme 1 by *S*_2_, which creates an indirect route of interaction between P and its supply block via reaction 3. **f** The rate characteristic plot for the supply and demand blocks of the intermediate P in (**e**). In (**d**) and (**f**) only the total and partial response coefficients of the supply block towards *p* are shown and the slopes of the partial response coefficients (*solid lines*) add up to that of the total response coefficient (*dashed line*)

Generalised supply-demand analysis (GSDA) is an extension of metabolic supply-demand analysis [13]. In GSDA a metabolic pathway is partitioned into reaction blocks around each variable metabolite, as shown for the metabolite P in the linear pathway in Fig. 1a. The producing and consuming blocks of this intermediate are termed the supply and demand blocks, respectively. The concentration of each variable is fixed and, in turn, varied over a range to generate combined rate characteristic plots [14] of the supply and demand blocks linked to the metabolite as shown in Fig. 1b. The response coefficients of the supply and demand blocks towards the linking metabolite are calculated, along with the elasticity coefficients of the reactions in these blocks that are directly connected to the intermediate (i.e., the last reaction in the supply block and the first in the demand block).

This approach can be used to determine which block has the most control over the system flux and which block determines the degree of homoeostasis of the linking intermediate by comparing the response coefficients (or gradients of the rate characteristics at steady-state) of the supply and demand blocks towards the intermediate. In the case where

(6) $$ \left|\frac{R^{J_{\text{supply}}}_{p}}{R^{J_{\text{demand}}}_{p}}\right| > 1  $$

as in Fig. 1b, the flux is predominantly controlled by the demand block, while in the opposite case where the ratio is <1, the flux is controlled by the supply. The degree of homoeostatic maintenance of the P concentration (denoted by *p*) depends on the value of *RpJ*_demand_−*RpJ*_supply_; the larger this value the smaller the absolute value of the concentration-control coefficients of the supply and demand blocks on *p*, and the better its homoeostasis [13, 15].

### Multiple routes of interaction

GSDA can also be used to identify the different routes of interaction between an intermediate and a reaction block and to quantify the relative contribution of each of these routes to the total response. In Fig. 1c an additional interaction of P with its supply block occurs through the allosteric inhibition of reaction 1 by P. The flux-response coefficient of the supply block to P is now the sum of two terms, called partial response coefficients:

(7) $$ \begin{aligned} R^{J_{\text{supply}}}_{p} &= \,{~\!\!}^{v_{1}}R^{J_{\text{supply}}}_{p} + \,{~\!\!}^{v_{2}}R^{J_{\text{supply}}}_{p} \\ &= C^{J_{\text{supply}}}_{v_{2}}\varepsilon^{v_{2}}_{p} + C^{J_{\text{supply}}}_{v_{1}}\varepsilon^{v_{1}}_{p} \end{aligned}  $$

According to the partitioned-response property (Eq. 5), each partial response coefficient is the product of an elasticity coefficient and a control coefficient. These control coefficients represent the sensitivities of flux local to the supply block, and must be distinguished from the flux-control coefficients of the full supply-demand system, i.e., $C^{J_{\text {supply}}}_{v_{i}} \neq C^{J}_{v_{i}}$. The partial responses can be represented visually in the form of a rate characteristic plot as shown in Fig. 1d.

Whereas in Figs. 1a and c the metabolite P is the sole link between the supply and demand blocks, it is of course also possible, as in Fig. 1e, for an intermediate within one reaction block to provide a link to the other reaction block. Here, reaction 1 is inhibited by S_2_, so that a change in P will be transmitted via reaction 3 in the demand block to S_2_, affecting its concentration, which, in turn, affects reaction 1. The overall effect (as shown in Fig. 1f) is similar to that in Figs. 1b and d, except that P also affects the supply block indirectly via S_2_. This indirect effect of P via S_2_ is made explicit in the second right-hand term of the following expression:

(8) $$ \begin{aligned} R^{J_{\text{supply}}}_{p} &= \,{~\!\!}^{v_{1}}R^{J_{\text{supply}}}_{p} + \,{~\!\!}^{v_{3}}R^{J_{\text{supply}}}_{p} \\ &= C^{J_{\text{supply}}}_{v_{2}}\varepsilon^{v_{2}}_{p} + C^{J_{\text{supply}}}_{v_{3}}\varepsilon^{v_{3}}_{p} \\ &= C^{J_{\text{supply}}}_{v_{2}}\varepsilon^{v_{2}}_{p} + C^{J_{\text{supply}}}_{v_{1}} \varepsilon^{v_{1}}_{S_{2}} C^{S_{2}}_{v_{3}} \varepsilon^{v_{3}}_{p} \end{aligned}  $$

The fixed metabolite can also interact with a particular reaction block through indirect stoichiometric linkages. In this case a change in the fixed metabolite concentration is transmitted via one reaction block to another through stoichiometric connections in the rest of the network, in a similar manner to the previously described allosteric interaction (Fig. 1e). The difference here is that, instead of an allosteric interaction, metabolites and reactions can link both reaction blocks via a stoichiometric route that does not involve the fixed metabolite. The members of a moiety-conserved cycle (discussed below) are an example of intermediates that can link reaction blocks in this way because of their stoichiometric involvement in numerous reactions at various points in the network.

### Moiety-conserved cycles

Moiety-conserved cycles require special consideration in GSDA as the total concentration of the members of the conserved cycle must remain constant. The individual member concentrations are therefore not free to vary in the same way as other metabolites.

In order to perform parameter scans on the members of the moiety-conserved cycles without breaking moiety conservation, the individual members of a cycle can be substituted with a single metabolite representing their ratio to one another. The concentrations of the members of each cycle are calculated using the total moiety concentration and the value of the ratio. Using the ATP/ADP moiety-conserved cycle as an example, with *ϕ*_*A*_ representing the ratio of ATP to ADP and *C*_*A*_ the total moiety concentration, the equations below illustrate this principle:

$$ \begin{aligned} {\phi_{A}} &= \frac{[\text{ATP}]}{[\text{ADP}]} \\ C_{A} &=\ \ [\text{ATP}] + [\text{ADP}] \\  \therefore [\text{ATP}] &= \frac{{\phi_{A}} \cdot C_{A}}{1+ {\phi_{A}}} \quad\text{and}\quad [\text{ADP}] = \frac{C_{A}}{1 + {\phi_{A}}} \end{aligned}  $$

### Software

The Python simulator for cellular systems (PySCeS) [16] together with the RateChar [8] module that forms part of the PySCeSToolbox package (https://github.com/PySCeS/PyscesToolbox) was used to perform the modelling experiments and metabolic control analysis and to generate the resulting rate characteristic plots. RateChar automatically performs supply-demand analysis and produces rate-characteristic plots for each metabolite in a metabolic model.

### Models

#### Pyruvate branch metabolism

**Fig. 2:**
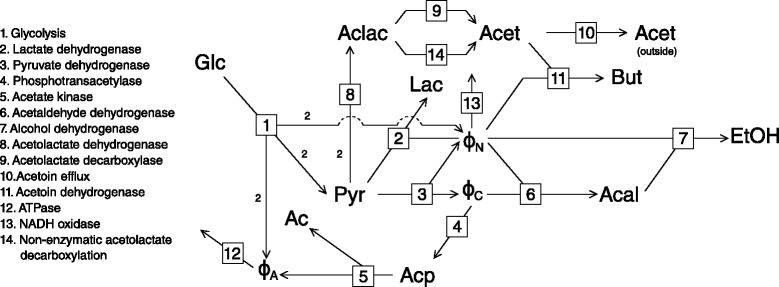
The pyruvate branch pathway as defined by Hoefnagel et al. [11]. Reactions are numbered according to the key. The stoichiometry of each reaction is 1 to 1, except for reaction 1 where *G* *l* *c*+2*A* *D* *P*+2*N* *A* *D* ^+^→2Pyr+2ATP+2NADH and reaction 8 where $\mathrm {2Pyr \rightleftharpoons Aclac}$. Intermediates are abbreviated as follows: Ac: acetate; Acal: acetaldehyde; Acet: acetoin; Aclac: acetolactate; Acp: acetyl phosphate; Glc: glucose; Lac: lactate; But: 2,3-butanediol; Pyr: pyruvate; EtOH: ethanol

To investigate the effects of multiple routes of regulation through moiety-conserved cycles we used a kinetic model of pyruvate metabolism in lactic acid bacteria. The model was originally constructed by Hoefnagel et al. [11] and retrieved from JWS online [5] in the PySCeS model descriptor language format [16, 17]. The structure of the model is outlined in Fig. 2. This model was chosen for our investigation on the basis of its three different moiety-conserved cycles that interact with a variety of reactions across different branches in the pathway.

The members of the ATP/ADP, acetyl-CoA/CoA and NADH/NAD ^+^ conserved moieties were treated as outlined previously, with the symbols *ϕ*_*A*_,*ϕ*_*C*_ and *ϕ*_*N*_ representing the metabolite ratios. The values of *ϕ*_*A*_ and *ϕ*_*N*_ were fixed and varied over the ranges shown in Table 1. Together with the results from metabolic control analysis these data were used to generate Figs. 4, 5, 6, 7 and 8.

**Table 1:** Pyruvate metabolism model scan ranges

Metabolite	Scan range	Steady-state ratio
*ϕ*_*A*_	0.06–457.51	5.08
*ϕ*_*N*_	0.0002–1.77	0.02

The ranges over which the variable metabolite concentration ratios of the pyruvate metabolism model [11] were varied to generate Figs. 4, 5, 6, 7 and 8

#### Aspartate metabolism

**Fig. 3:**
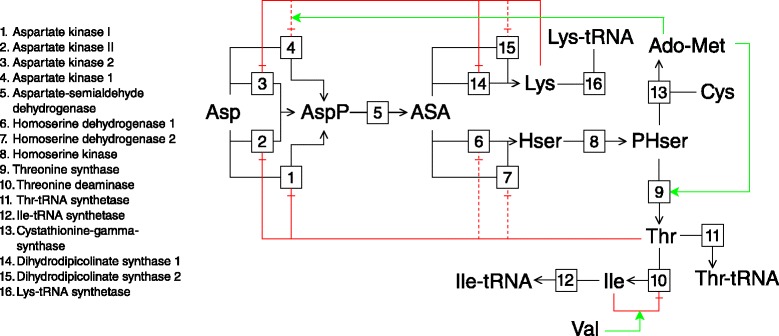
The aspartate-derived amino acid synthesis pathway as defined by Curien et al. [12]. Reactions are numbered according to the key. Green lines ending with solid arrows indicate activation of a reaction or potentiation of an allosteric effect, while red lines ending with daggers indicate inhibition of reactions or damping of an allosteric effect. Strong allosteric effects are indicated with solid lines, while weak effects are shown with dashed lines. The stoichiometry of each reaction is 1 to 1. Intermediates are abbreviated as follows: Ado-Met: S-adenosylmethionine; ASA: aspartate-semialdehyde; Asp: aspartate; AspP: aspartyl phosphate; Cys: cysteine; Hser: homoserine; Ile: isoleucine; Lys: lysine; PHser: phosphohomoserine; Thr: threonine; Val: valine

A kinetic model of aspartate-derived amino acid synthesis in *Arabidopsis* was used to investigate the effects of multiple routes of regulation brought about by allosteric effectors and multiple isoenzymes. The model was originally constructed by Curien et al. [12] in Berkeley Madonna format and was translated to the PySCeS MDL format. The structure of this pathway is outlined in Fig. 3.

**Fig. 4:**
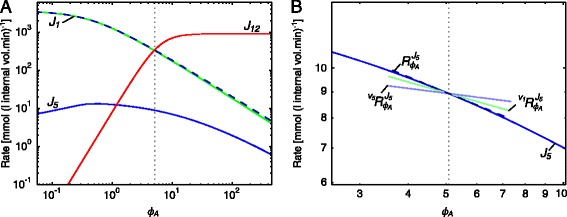
Rate characteristic plots of the reaction blocks of *ϕ*_*A*_ in the pyruvate branch model. **a** The fluxes of the demand block 12, and the supply blocks 1 and 5 of *ϕ*_*A*_. The unlabelled dashed curve represents the total supply. **b** The rate characteristic plot of the *ϕ*_*A*_-supply block 5 with partial and total response coefficients indicated as lines intersecting the *J*_5_-curve at the steady-state value of *ϕ*_*A*_. Partial response coefficients (*solid lines*) indicate the relative contribution of each route of interaction of *ϕ*_*A*_ with block 5 towards the total response coefficient (*dashed line*). The steady-state value of *ϕ*_*A*_ is indicated as a vertical dotted line in both (**a**) and (**b**) (see Table 1)

**Fig. 5:**
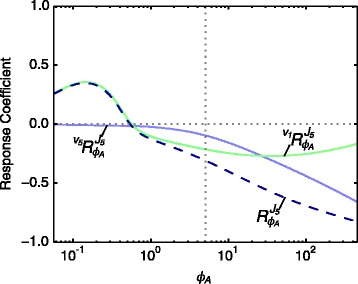
Partial and total response coefficients of *J*_5_ towards *ϕ*_*A*_ as a function of *ϕ*_*A*_. Partial response coefficients (*solid lines*) indicate the relative contribution of each route of interaction of *ϕ*_*A*_ with reaction block 5 towards the total response coefficient (*dashed line*) over the *ϕ*_*A*_-range as indicated in Table 1. The steady-state value of *ϕ*_*A*_ is indicated as a vertical dotted line

**Fig. 6:**
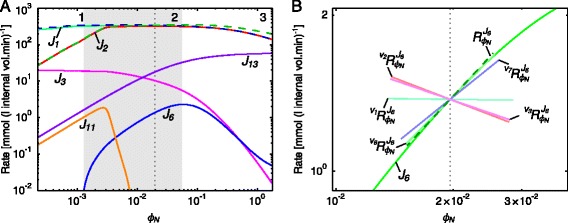
Rate characteristic plots of the reaction blocks of *ϕ*_*N*_ in the pyruvate branch model. **a** The fluxes of the demand blocks 2, 6, 11 and 13, and the supply blocks 1 and 3 of *ϕ*_*N*_. The unlabelled dashed curves represent the total supply (*blue*) and demand (*green*). **b** The rate characteristic plot of the *ϕ*_*N*_-demand block 6 with partial and total response coefficients indicated as lines intersecting the *J*_6_-curve at the steady-state value of *ϕ*_*N*_. Partial response coefficients (*solid lines*) indicate the relative contribution of each respective route of interaction of *ϕ*_*N*_ with the reaction block 6 towards the total response coefficient (*dashed line*). Note that $\protect \phantom {\dot {i}\!}{{~\!\!}^{v_{11}}{R^{J_{6}}_{\phi _{N}}}}$ and $\protect \phantom {\dot {i}\!}{{~\!\!}^{v_{13}}{R^{J_{6}}_{\phi _{N}}}}$ were omitted due to their zero contributions towards $\protect \phantom {\dot {i}\!}{R^{J_{6}}_{\phi _{N}}}$. The steady-state value of *ϕ*_*N*_ is indicated as a vertical dotted line in both (**a**) and (**b**) (see Table 1)

**Fig. 7:**
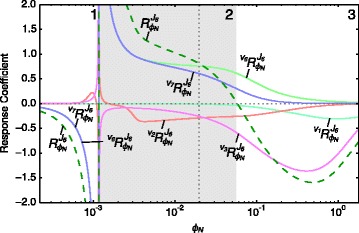
Partial and total response coefficients of *J*_6_ towards *ϕ*_*N*_ as a function of *ϕ*_*N*_. Partial response coefficients (*solid lines*) indicate the relative contribution of each respective route of interaction of *ϕ*_*N*_ with reaction block 6 towards the total response coefficients (*dashed lines*) over the *ϕ*_*N*_-range as indicated in Table 1. The steady-state value of *ϕ*_*N*_ is indicated as a vertical dotted line. Note that $\protect \phantom {\dot {i}\!}{{~\!\!}^{v_{11}}R^{J_{6}}_{\phi _{N}}}$ and $\protect \phantom {\dot {i}\!}{{~\!\!}^{v_{13}}R^{J_{6}}_{\phi _{N}}}$ were omitted due to their zero contributions towards $\protect \phantom {\dot {i}\!}{R^{J_{6}}_{\phi _{N}}}$

**Fig. 8:**
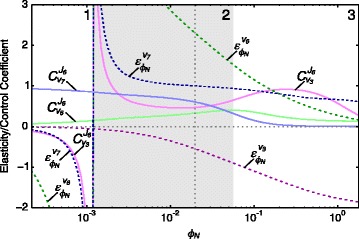
The most significant partial response coefficients contributing towards ${R^{J_{6}}_{\phi _{N}}}$ separated into elasticity and control coefficients. Elasticity coefficients (*dashed lines*) and control coefficients (*solid lines*) that make up the partial response coefficients of Fig. 7 are shown. Here $\protect \phantom {\dot {i}\!}{C^{J_{6}}_{v_{6}}} {\varepsilon ^{v_{6}}_{\phi _{N}}} = {{~\!\!}^{v_{6}}R^{J_{6}}_{\phi _{N}}}$, $\protect \phantom {\dot {i}\!}{C^{J_{6}}_{v_{7}}} {\varepsilon ^{v_{7}}_{{\phi _{N}}}} = {{~\!\!}^{v_{7}}R^{J_{6}}_{{\phi _{N}}}}$ and $\protect \phantom {\dot {i}\!}{C^{J_{6}}_{v_{3}}} {\varepsilon ^{v_{3}}_{{\phi _{N}}}} = {{~\!\!}^{v_{3}}R^{J_{6}}_{{\phi _{N}}}}$. The steady-state value of *ϕ*_*N*_ is indicated as a vertical dotted line (see Table 1)

**Fig. 9:**
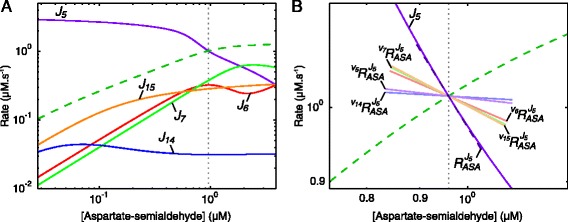
Rate characteristic plots of the reaction blocks of aspartate-semialdehyde in the aspartate metabolism model. **a** The fluxes of the demand blocks 6, 7, 14 and 15, and the supply block 5 of ASA. **b** The rate characteristic plot of the ASA-supply block 5 with partial and total response coefficients indicated as lines intersecting the *J*_5_-curve at the steady-state value of ASA. Partial response coefficients (*solid lines*) indicate the relative contribution of each respective route of interaction of ASA with block 5 towards the total response coefficient (*dashed line*). The unlabelled dashed curve in both (**a**) and (**b**) represents the total demand and the steady-state value of ASA is indicated as a vertical dotted line (see Table 2)

**Fig. 10:**
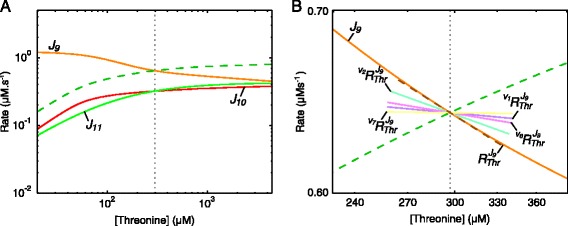
Rate characteristic plots of the reaction blocks of threonine in the aspartate metabolism model. **a** The fluxes of the demand blocks 10 and 11, and the supply block 9 of Thr. **b** The rate characteristic plot of the Thr-supply block 9 with partial and total response coefficients indicated as lines intersecting the *J*_9_-curve at the steady-state value of Thr. Partial response coefficients (*solid lines*) indicate the relative contribution of each respective route of interaction of Thr with reaction block 9 towards the total response coefficient (*dashed line*). Note that $\protect \phantom {\dot {i}\!}{{~\!\!}^{v_{9}}R^{J_{9}}_{\textit {Thr}}}$, $\protect \phantom {\dot {i}\!}{{~\!\!}^{v_{10}}R^{J_{9}}_{\textit {Thr}}}$ and $\protect \phantom {\dot {i}\!}{{~\!\!}^{v_{11}}R^{J_{9}}_{\textit {Thr}}}$ were omitted due to their zero contributions towards $\protect \phantom {\dot {i}\!}{R^{J_{9}}_{\textit {Thr}}}$. The unlabelled dashed curve in both (**a**) and (**b**) represents the total demand and the steady-state value of Thr is indicated as a vertical dotted line (see Table 2)

**Fig. 11:**
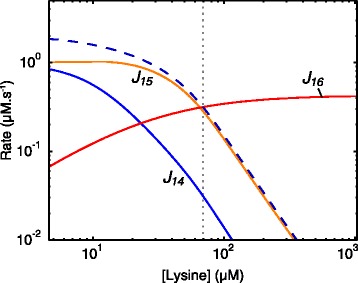
Rate characteristic plot showing the fluxes of the reaction blocks of lysine in aspartate metabolism. The demand block 16 and supply blocks 14 and 15 are indicated as solid lines. The unlabelled dashed curve represents the total supply. The steady-state value of Lys is indicated as a vertical dotted line (see Table 2)

**Fig. 12:**
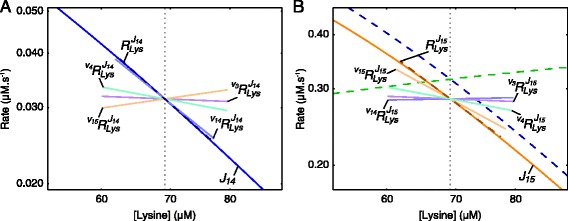
Rate characteristic plots of the supply blocks of lysine in the aspartate metabolism model. Partial and total response coefficients are indicated as lines intersecting the **a** *J*_14_-curve and **b** *J*_15_-curve at the steady-state value of Lys (see Table 2). Partial response coefficients (*solid lines*) indicate the relative contribution of each respective route of interaction of Lys with reaction blocks 14 and 15 towards the total responses (*dashed lines*) at the steady-state. The unlabelled dashed curves represent the total supply (*blue*) and total demand (*green*). The steady-state value of Lys is indicated as a vertical dotted line in both (**a**) and (**b**)

**Fig. 13:**
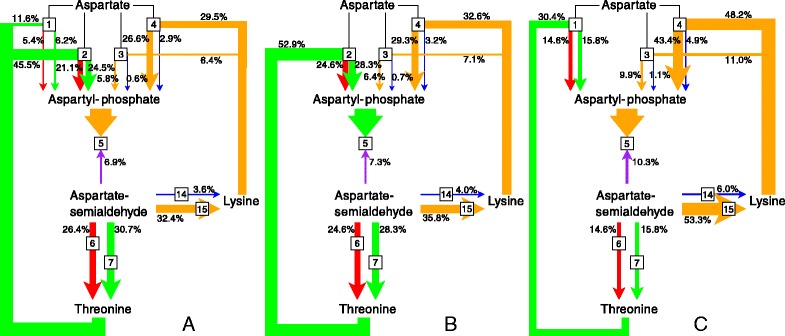
The importance of the various routes of regulation of ASA with its supply block. **a** The reference model. **b** AKI knockout. **c** AKII knockout. Knockouts in (**b**) and (**c**) were performed by respectively setting the concentrations of AKI and AKII to zero. All models were at steady-state (see Table S2 in Additional file 6)

For this case study the focus was to identify and quantify the different routes of regulation of aspartate-semialdehyde (ASA) with its supply block for the wild type as well as for knockouts of AKI and AKII. ASA, lysine (Lys) and threonine (Thr) were fixed and varied over the ranges shown in Table 2 and used, together with results from MCA, to generate Figs. 9, 10, 11, 12 and 13.

**Table 2:** Aspartate metabolism model scan ranges

Metabolite	Scan range (*μ* *M*)	Steady-state conc. (*μ* *M*)
ASA	0.024–32.97	0.96
Thr	19.80–4453.93	296.93
Lys	4.61–1037.38	69.16

The ranges over which the variable metabolite concentrations of the aspartate-derived amino acid synthesis pathway model [12] were varied to generate Figs. 9, 10, 11 and 12

The knockouts of AKI, AKII and both AKI and AKII were simulated by setting their respective enzyme concentrations to zero in the model.

#### Model code

Code for both models is provided in PySCeS model descriptor language (Additional files 1 and 2) and in the standard Systems Biology Markup Language (SBML) format [18] (Additional files 3 and 4). An IPython notebook containing instructions and scripts to reproduce the analyses from this paper is provided as Additional file 5.

## Results

### Regulatory connections via moiety-conserved cycles

The pyruvate branch model in Fig. 2 contains three moiety conserved cycles, ATP/ADP, NADH/NAD ^+^ and acetyl-CoA/CoA, with their members modelled as variable species. These species take part in a number of reactions across the three main branches leading from pyruvate, thereby enabling the branches to communicate with each other. This model is therefore an ideal candidate for investigating the type of behaviour that can occur due to the presence of moiety-conserved cycles in general, and more specifically due to the ATP/ADP and NADH/NAD ^+^ cycles, which are ubiquitous in metabolism. In this section we show how the presence of ATP/ADP and NADH/NAD ^+^ causes unexpected and non-monotonic flux response behaviour.

#### Regulatory routes of ATP/ADP

The first conserved moiety we shall investigate is ADP, due to its relatively small number of interactions in this pathway. The ‘metabolite’ *ϕ*_*A*_ represents the ratio of ATP to ADP. An increase in *ϕ*_*A*_ implies an increase in ATP concentration and a concomitant decrease in ADP concentration within the constraint of their sum being constant. In this pathway *ϕ*_*A*_ is produced by acetate kinase (reaction 5) and a lumped glycolysis pathway (reaction 1), and is consumed by ATPase (reaction 12). The supply and demand blocks for *ϕ*_*A*_ are named according to the numbering of these consuming and producing reactions, i.e., block 1 and block 5 are *ϕ*_*A*_ supply blocks, while block 12 is a *ϕ*_*A*_ demand block, with the fluxes of these blocks symbolised by *J*_1_, *J*_5_ and *J*_12_ respectively (this naming convention for reaction blocks and their corresponding fluxes is used throughout this paper).

The rate characteristic plot (Fig. 4a) shows the effect of a change in *ϕ*_*A*_ on its supply and demand blocks. In steady-state all the reaction blocks respond as expected towards increasing *ϕ*_*A*_, with decreases in *J*_1_ and *J*_5_ and an increase in *J*_12_. At *ϕ*_*A*_ values below 0.4, however, there was an *increase* in *J*_5_ in response to increasing product (ATP) and decreasing substrate (ADP) concentration. Considering that acetate kinase is not product-activated, this positive flux response was unexpected.

The source of the flux response of block 5 towards *ϕ*_*A*_ was investigated using partial response coefficients (Fig. 4b) where a rate characteristic plot revealed that two different routes of interaction are responsible for the behaviour of this reaction block. At the steady-state, the partial response coefficients representing the relative importance of these two routes are both negative, leading to the observed negative total response coefficient of *J*_5_. The first partial response ($\phantom {\dot {i}\!}{{~\!\!}^{v_{5}}R^{J_{5}}_{{\phi _{A}}}}$) is due to the direct interaction of *ϕ*_*A*_ with block 5 via reaction 5. The second, and more significant, partial response ($\phantom {\dot {i}\!}{{~\!\!}^{v_{1}}R^{J_{5}}_{\phi _{A}}}$) is due to the interaction of *ϕ*_*A*_ with reaction 1, which also has a negative elasticity towards *ϕ*_*A*_ and forms part of both reaction blocks 1 and 5. The negative effect on *J*_5_ due to the decrease in *J*_1_ is a result of the flux relationships between these reaction blocks where a decrease in pyruvate production by block 1 leads to a decrease in flux of all pyruvate consuming reactions.

When considering the partial response coefficients over the complete range of *ϕ*_*A*_-values (Fig. 5), we saw that whereas $\phantom {\dot {i}\!}{{~\!\!}^{v_{5}}R^{J_{5}}_{\phi _{A}}}$ is close to zero for *ϕ*_*A*_-values below 0.4, $\phantom {\dot {i}\!}{{~\!\!}^{v_{1}}R^{J_{5}}_{\phi _{A}}}$ is positive, thereby being solely responsible for the observed increase in *J*_5_ over this range of *ϕ*_*A*_-values. This positive response can again be traced to the flux relationships between *J*_1_ and *J*_3_, but in this case *J*_2_ and *J*_8_ also play a role. While *J*_1_ does decrease, the fluxes *J*_2_ and *J*_8_ decrease even more, resulting in an increase in *J*_3_. Additionally, a decrease in *J*_6_ causes flux to be diverted to *J*_4_. Both these effects lead to the observed increase in *J*_5_ for this *ϕ*_*A*_-range (Fig. 4a).

These results indicate that the indirect route of interaction of *ϕ*_*A*_ with block 5 plays a large role in the regulation of the flux through this block, and is indeed the most prominent regulatory route for *ϕ*_*A*_-values below 30.

#### Regulatory routes of NADH/NAD ^+^

While notable, the counter-intuitive response to *ϕ*_*A*_ is brought about by only two partial responses due to ATP/ADP acting as an intermediate in only a few reactions in the pathway. The NAD ^+^ moiety, on the other hand, interacts with more reaction blocks than either of the CoA and ADP moieties: *ϕ*_*N*_ is produced by a lumped glycolysis pathway (reaction 1) and pyruvate dehydrogenase (reaction 3), and consumed by lactate dehydrogenase (reaction 2), acetoin dehydrogenase (reaction 11), acetaldehyde dehydrogenase (reaction 6), alcohol dehydrogenase (reaction 7) and NADH oxidase (reaction 13). While block 6 and block 7 are separate demand blocks for *ϕ*_*N*_, they are also linked by acetaldehyde and therefore their rates are equal at steady-state. In cases where the observed results for these blocks are identical we refer only to block 6 for the sake of brevity. Due to the numerous interactions of *ϕ*_*N*_ in this system, there is potential for complex flux response behaviour.

The flux of reaction block 6, *J*_6_ responds non-monotonically to changing *ϕ*_*N*_ (Fig. 6a), in contrast to the fluxes of the other blocks that *ϕ*_*N*_ reacts with, which respond monotonically as anticipated. In the *ϕ*_*N*_-range below 0.0012 *J*_6_ is negative (which implies that reaction 6 in Fig. 2 proceeds in the reverse direction), but becomes less negative as *ϕ*_*N*_ increases; at *ϕ*_*N*_= 0.0012 block 6 is at equilibrium and *J*_6_=0. In the range 0.0012<*ϕ*_*N*_<0.057*J*_6_ is positive and increases to a maximum at *ϕ*_*N*_=0.057. In the *ϕ*_*N*_-range above 0.057 *J*_6_ decreases monotonically. These three *ϕ*_*N*_-ranges will henceforth be referred to as range 1, 2, and 3 respectively.

As before, partial response coefficients explain the behaviour of reaction block 6. At the steady-state, four routes of interaction of *ϕ*_*N*_ with block 6 contribute significantly to the total response ${R^{J_{6}}_{\phi _{N}}}$, as shown in the rate characteristic plot in Fig. 6b. The direct interactions via reaction 6 and reaction 7 result in positive partial responses ($\phantom {\dot {i}\!}{{~\!\!}^{v_{6}}R^{J_{6}}_{\phi _{N}}}$ and $\phantom {\dot {i}\!}{{~\!\!}^{v_{7}}R^{J_{6}}_{\phi _{N}}}$) due to *ϕ*_*N*_ acting as a substrate for these reactions. On the other hand, the interactions via reactions 2 and 3, represented by $\phantom {\dot {i}\!}{{~\!\!}^{v_{2}}R^{J_{6}}_{\phi _{N}}}$ and $\phantom {\dot {i}\!}{{~\!\!}^{v_{3}}R^{J_{6}}_{\phi _{N}}}$, affect *J*_6_ negatively; by decreasing *J*_3_ via reactions 2 and 3, *ϕ*_*N*_ decreases *ϕ*_*C*_, thereby limiting the availability of this additional substrate for reaction 6.

The source of the non-monotonic behaviour of block 6 becomes clear when the partial response coefficients of *J*_6_ towards a range of *ϕ*_*N*_-values are computed (Fig. 7). The non-monotonic total response coefficient (the dashed green line) is the sum of multiple partial response coefficients which are themselves non-monotonic, their contributions to the total varying greatly over the *ϕ*_*N*_-range. There is a singularity at *ϕ*_*N*_=0.0012 between ranges 1 and 2 which correlates with the equilibrium state that block 6 goes through when *J*_6_ changes direction.

In ranges 1 and 2 the total response coefficient behaviour is determined mostly by $\phantom {\dot {i}\!}{{~\!\!}^{v_{6}}R^{J_{6}}_{\phi _{N}}}$ and $\phantom {\dot {i}\!}{{~\!\!}^{v_{7}}R^{J_{6}}_{\phi _{N}}}$ as the values of the other partial response coefficients are low and undergo little change. In range 3, however, a slightly more complex interplay of effects brings about total response behaviour. Here the decline of the total response coefficient and its subsequent reversal of sign was caused by the increase in magnitude of the negative partial response coefficient $\phantom {\dot {i}\!}{{~\!\!}^{v_{3}}R^{J_{6}}_{\phi _{N}}}$ and the decrease in magnitude of $\phantom {\dot {i}\!}{{~\!\!}^{v_{6}}R^{J_{6}}_{\phi _{N}}}$ and $\phantom {\dot {i}\!}{{~\!\!}^{v_{7}}R^{J_{6}}_{\phi _{N}}}$.

By separating the partial response coefficients into their elasticity and control coefficient components according to the partitioned response equation (Eq. 5), we obtain a clearer view of the role of local versus systemic effects in bringing about the flux response. The control and elasticity coefficients that make up $\phantom {\dot {i}\!}{{~\!\!}^{v_{6}}R^{J_{6}}_{\phi _{N}}}$, $\phantom {\dot {i}\!}{{~\!\!}^{v_{7}}R^{J_{6}}_{\phi _{N}}}$ and $\phantom {\dot {i}\!}{{~\!\!}^{v_{3}}R^{J_{6}}_{\phi _{N}}}$, i.e, the partial responses that make the largest contribution to ${R^{J_{6}}_{\phi _{N}}}$, are shown in Fig. 8 (see legend for their partitioned response equations). Since elasticity coefficients tend to infinity at equilibrium, $\phantom {\dot {i}\!}{\varepsilon ^{v_{5}}_{\phi _{N}}}$ and $\phantom {\dot {i}\!}{\varepsilon ^{v_{7}}_{\phi _{N}}}$ largely determine $\phantom {\dot {i}\!}{{~\!\!}^{v_{5}}R^{J_{5}}_{\phi _{N}}}$ and $\phantom {\dot {i}\!}{{~\!\!}^{v_{7}}R^{J_{6}}_{\phi _{N}}}$ around *ϕ*_*N*_=0.0012. In range 2, both $\phantom {\dot {i}\!}{\varepsilon ^{v_{6}}_{\phi _{N}}}$ and $\phantom {\dot {i}\!}{\varepsilon ^{v_{7}}_{\phi _{N}}}$ indicate that neither reaction 6 nor 7 has reached saturation. At the end of range 3 $\phantom {\dot {i}\!}{\varepsilon ^{v_{7}}_{\phi _{N}}}$ still has a significant positive value, while $\phantom {\dot {i}\!}{\varepsilon ^{v_{6}}_{\phi _{N}}}$ has declined to nearly zero (indicating that reaction 6 is far from equilibrium and close to full saturation). For the control coefficients $\phantom {\dot {i}\!}{C^{J_{6}}_{v_{6}}}$ and $\phantom {\dot {i}\!}{C^{J_{6}}_{v_{7}}}$, the situation is somewhat reversed. Here, while having significantly lower values than their corresponding elasticity coefficients for most *ϕ*_*N*_-values, the decline in $\phantom {\dot {i}\!}{C^{J_{6}}_{v_{7}}}$ is much more dramatic than that of $\phantom {\dot {i}\!}{C^{J_{6}}_{v_{6}}}$. At *ϕ*_*N*_ =0.2, $\phantom {\dot {i}\!}{C^{J_{6}}_{v_{7}}}$ is nearly zero, while at the highest tested *ϕ*_*N*_-value $\phantom {\dot {i}\!}{C^{J_{6}}_{v_{6}}}$ and $\phantom {\dot {i}\!}{\varepsilon ^{v_{6}}_{\phi _{N}}}$ are nearly equal. These results indicate that the decline of the partial responses $\phantom {\dot {i}\!}{{~\!\!}^{v_{6}}R^{J_{6}}_{\phi _{N}}}$ and $\phantom {\dot {i}\!}{{~\!\!}^{v_{7}}R^{J_{6}}_{\phi _{N}}}$ at higher *ϕ*_*N*_-values is mostly due to the decline in control of *v*_7_ on *J*_6_ for $\phantom {\dot {i}\!}{{~\!\!}^{v_{7}}R^{J_{6}}_{\phi _{N}}}$ and the decline in elasticity of *v*_6_ towards *ϕ*_*N*_ for $\phantom {\dot {i}\!}{{~\!\!}^{v_{6}}R^{J_{6}}_{\phi _{N}}}$. For $\phantom {\dot {i}\!}{{~\!\!}^{v_{3}}R^{J_{6}}_{\phi _{N}}}$, both $\phantom {\dot {i}\!}{C^{J_{6}}_{v_{3}}}$ and $\phantom {\dot {i}\!}{\varepsilon ^{v_{3}}_{\phi _{N}}}$ contribute to the declining negative partial response coefficient. We saw, however, that the inflection point observed in $\phantom {\dot {i}\!}{{~\!\!}^{v_{3}}R^{J_{6}}_{\phi _{N}}}$ at *ϕ*_*N*_=0.4 (Fig. 7) is due to the contribution of the control coefficient rather than that of the elasticity coefficient.

### Regulatory connections via feedback and isoenzymes

The aspartate-derived amino acid synthesis pathway model in Fig. 3 contains a number of features that allow for multiple routes of regulation. Three of the steps are catalysed by isozymes that are allosterically modified by a variety of pathway intermediates and the pathway has multiple branch points. The isoenzymes also differ in terms of their kinetic properties and therefore respond differently to changes in the concentrations of their effectors. In this section we explore the importance of various routes of regulation of one intermediate with its supply block and elucidate the roles the various isoenzymes play within these routes.

#### Routes of interaction through different allosteric feedbacks

The first branch point of the aspartate-derived amino acid synthesis pathway occurs at aspartate-semialdehyde (ASA), which is produced by aspartate-semialdehyde dehydrogenase (ASADH or reaction 5) and consumed by two separate metabolic branches. The first step of the branch that produces threonine (Thr), cysteine (Cys) and isoleucine (Ile) as end products is catalysed by two isoenzymes, homoserine dehydrogenase I and II (HSDHI and HSDHII; reactions 6 and 7). The first step in the branch that produces lysine (Lys) as an end product is catalysed by the isoenzymes dihydrodipicolinate synthase 1 and 2 (DHDPS1 and DHDPS2; reactions 14 and 15). Thr and Lys inhibit the isoenzymes catalysing the first step of their respective branches, as well as two of the four aspartate kinase isoenzymes catalysing the first step in the pathway: Thr inhibits aspartate kinase I and II (AKI and AKII; reactions 1 and 2) and Lys inhibits aspartate kinase 1 and 2 (AK1 and AK2; reactions 4 and 3). Demand blocks of ASA can be defined according to the four consuming enzymes, with fluxes *J*_14_ and *J*_15_ in the Lys-branch, and *J*_6_ and *J*_7_ in the Thr-branch. Alternatively, we can define the demand blocks according to the two separate metabolic branches where *J*_6_+*J*_7_=*J*_8_ for the Thr branch and *J*_14_+*J*_15_=*J*_16_ for the Lys branch. The rate characteristic plot shown in Fig. 9a shows that at steady-state most of the flux proceeds towards Thr production with *J*_6_ and *J*_7_ being nearly equal. The flux towards Lys is carried mostly by block 15.

The inhibition of the AK isoenzymes by Thr and Lys enables changes in ASA to be transmitted to its supply block via these intermediates, which occur in the ASA-demand blocks. Partial response coefficients were used to determine the contribution of each of these routes of interaction to ${R^{J_{6}}_{\textit {ASA}}}$ and therefore to quantify their importance in regulating *J*_5_ (Fig. 9b). Because of the very low degree of control of reaction 5 over its own flux, the direct interaction of ASA with block 5 via reaction 5 makes the second smallest contribution towards the total response despite its relatively high elasticity towards ASA (see Table S1 of Additional file 6). Instead the interactions of ASA with block 6, block 7 and block 15 contribute the most towards the observed total response. The enzymes catalysing the first steps of these blocks (reactions 6, 7 and 15) have lower sensitivities towards ASA than reaction 5, but much more control over *J*_5_. The isoenzymes in each branch have practically identical elasticity coefficients towards ASA at the steady-state, therefore the difference in responses between the individual reactions in each branch is due to the differences in the degree to which these reactions control *J*_5_, as determined by the flux carried by each reaction.

While the above results quantify the importance of each of the routes of ASA supply block regulation and show that inhibition of reactions 1–4 by Thr and Lys plays an important regulatory role, we still have to quantify the amount of regulation that takes place via each of these four AK isoenzymes. This can be achieved by quantifying the contribution of the regulatory routes of the supply blocks of Thr and Lys and combining these results with the previous results.

The rate characteristic plot shown in Fig. 10a illustrates the behaviour of the reaction blocks of Thr in response to changes in this metabolite’s concentration. Here the Thr supply block (block 9), which ends with the enzyme threonine synthase (reaction 9), also encompasses the ASA supply block. Figure 10b shows the partial responses of *J*_9_ towards Thr at the steady-state. It is clear that, as reaction 9 is insensitive towards its product Thr, the observed flux response is solely due to the inhibition of the upstream reactions 1, 2, 6 and 7 by Thr. In order to quantify the regulation of the ASA supply block, only the partial response coefficients of reaction block 9 towards Thr that represent routes passing through the ASA supply block, i.e., reactions 1 and 2, are of interest. We saw that despite only 1.8 and 9.4 % of total flux respectively passing through reactions 1 and 2, and despite the resulting small degree of control these reactions have of over *J*_9_, their high elasticities towards Thr cause both $\phantom {\dot {i}\!}{{~\!\!}^{v_{1}}R^{J_{9}}_{\textit {Thr}}}$ and $\phantom {\dot {i}\!}{{~\!\!}^{v_{2}}R^{J_{9}}_{\textit {Thr}}}$ to contribute significantly towards $R^{J_{9}}_{Thr}$. However, regardless of the specific contributions of these two routes in regulating *J*_9_, the proportion of each of $\phantom {\dot {i}\!}{{~\!\!}^{v_{1}}R^{J_{9}}_{\textit {Thr}}}$ and $\phantom {\dot {i}\!}{{~\!\!}^{v_{2}}R^{J_{9}}_{\textit {Thr}}}$ to their total indicate the proportion of ASA supply block flux regulation taking place through reactions 1 and 2 (Fig. 13a). Using the regulation of the ASA supply block by ASA via the route that passes through blocks 6 and 7 and subsequently through reaction 1 due to its inhibition by Thr as an example, the percentage regulation of this block taking place via reaction 1 (denoted by $\phantom {\dot {i}\!}{~\!\!}^{v_{1}}\chi ^{J_{5}}_{\textit {ASA}}$) can be calculated as follows:

(9) $$ {\fontsize{8.9pt}{9.3pt}\selectfont{\begin{aligned} {}{~\!\!}^{v_{1}}\chi^{J_{5}}_{ASA} &= \frac{{~\!\!}^{v_{6}}R^{J_{5}}_{ASA}+ \,{~\!\!}^{v_{7}}R^{J_{5}}_{ASA}}{R^{J_{5}}_{ASA}} \times \frac{{~\!\!}^{v_{1}}R^{J_{9}}_{Thr}} {{~\!\!}^{v_{1}}R^{J_{9}}_{Thr}+\,{~\!\!}^{v_{2}}R^{J_{9}}_{Thr}}\times100\\ &= \frac{(-0.276)+(-0.320)}{-1.043} \times \frac{-0.035}{(-0.035)+(-0.137)}\times100\\ &=11.628~\% \end{aligned}}}  $$

Lys is produced by reactions 14 and 15 and therefore has two supply blocks. In this model the multi-step process of converting ASA to Lys was combined into a single step due to the irreversibility of reactions 14 and 15 [12]. At the steady-state, block 15 carries much more flux towards Lys than block 14, which, as we will see, affects the regulation of these blocks via each other (Fig. 11). The partial responses of the two Lys supply blocks to Lys are shown in Fig. 12. In contrast to the regulation of the Thr supply block where inhibition of the AK isoenzymes by Thr is the most important regulatory route, inhibition of the initial step of the Lys branch (reactions 14 and 15) by Lys is more important in eliciting a response in each of *J*_14_ and *J*_15_ than is inhibition of the AK isoenzymes. Interestingly, the inhibition of reaction 15 by Lys results in a positive response in *J*_14_ due to the attenuation of competition for the substrate ASA. The mirror effect of Lys-inhibition of *J*_14_ on *J*_15_ is also observed, but is smaller, as *J*_14_ carries much less flux towards Lys; reaction 14 therefore has less control over *J*_15_ than reaction 15 has over *J*_14_. In the case of reactions 3 and 4, which are relevant to the routes of regulation of ASA with its supply block, we saw that reaction 4 contributes more towards the observed negative total response coefficients ${R^{J_{14}}_{\textit {Lys}}}$ and ${R^{J_{15}}_{\textit {Lys}}}$ than reaction 3 due to having significantly more control over *J*_14_ and *J*_15_ (see Table S1 of Additional file 6). The same technique used to elucidate the importance of reactions 1 and 2 in the regulation of the ASA supply block by ASA (as demonstrated in Eq. 9) was used here to determine the importance of the routes of regulation involving reactions 3 and 4 (Fig. 13a).

#### The effect of isoenzyme knockouts

The previous section showed the dissection of the routes of regulation of the ASA supply block rate by ASA for the wild type pathway. Using the same techniques, it was possible to gain insight into the regulation of this reaction block by ASA under alternative conditions. Here we performed the same analysis for knockout models of (1) AKI, (2) AKII, and (3) both AKI and AKII. These knockouts, among others, were previously modelled by Curien et al. [19]. Steady-state analysis was performed as shown in Table S2 of Additional file 6 and the quantification of the importance of the various routes of regulation of *J*_5_ by ASA is shown in Figs. 13b and c.

Together, reactions 1 and 2 contribute only 12 % of the total flux of the pathway at the reference steady-state (Table S2 of Additional file 6); however, as shown in the previous section, most of the regulation of the ASA supply block by ASA takes place via these two reactions. When taking all the AKs into consideration, reaction 2 is the most important and reaction 1 the third most important in terms of regulation. While the importance of the AK isoenzymes in terms of ASA supply flux regulation cannot be predicted by their flux contributions alone, there is nevertheless still a relationship between isoenzyme flux and regulatory importance because the degree of ASA supply flux control by the isoenzymes is a function of their relative flux contributions. Reaction 2 contributes 9.4 % of the total flux with 45.5 % of regulation taking place through it, while reaction 1 contributes 1.8 % of total flux with only 11.6 % of regulation taking place through this reaction (Fig. 13a).

The knockout models highlighted once more the disconnection between flux and regulatory importance. The AKI knockout causes less than a 1 % decrease in total flux (Table S2 of Additional file 6), with a concomitant decrease in regulation via the Thr inhibition route from 57.1 to 52.9 % (Fig. 13b). Here it was clear that while regulation via this route decreases, reaction 2 can compensate for the loss of reaction 1 by largely taking over its regulatory role. On the other hand, a knockout of reaction 2 causes a 3.7 % (Table S2 of Additional file 6) decrease in flux, which subsequently causes regulation via Thr inhibition to drop to 30.4 % (Fig. 13c). In spite of the regulatory importance of reaction 1 increasing by 162.1 % compared to the wild-type (in contrast to a 83.3 % increase in flux through this reaction), it cannot fully compensate for the loss of reaction 2. For all three knockout models, regulation is diverted mostly towards the Lys branch with 86.1 % of regulation occurring via this branch for the double knockout (not shown), once more indicating the relatively low importance of the direct route of interaction of ASA with its supply block.

In addition to affecting the relative importance of the routes of regulation of the ASA supply block, the isoenzyme knockouts also affect the magnitude of the ASA supply and demand block responses (Table 3). There is a decrease in ${R^{J_{5}}_{\textit {ASA}}}$ for each knockout model, with this effect being the least pronounced for the AKI knockout and the most pronounced for the double knockout. There is an increase in the Thr branch response (${R^{J_{8}}_{\textit {ASA}}}$) for the three knockouts, while the Lys branch response (${R^{J_{16}}_{\textit {ASA}}}$) remains relatively unchanged. These changes to the ratio of supply to demand response lead to changes in the functions of the reaction blocks in terms of flux control and ASA homoeostasis. At the reference state, both ASA demand branches have more control over their flux than the supply block, as indicated by the values of |*RASAJ*_5_/*RASAJ*_8_| and |*RASAJ*_5_/*RASAJ*_16_|>1 (Table 3). The decrease in ${R^{J_{5}}_{\textit {ASA}}}$ observed for the knockout models causes a decrease in the ratio of the supply response to the demand response, indicating that flux control shifts towards the supply block. For the double knockout, this shift causes a reversal of roles in terms of flux control where the supply block has more control of flux than the demand block.

**Table 3:** Analysis of the distribution of flux control between the supply and demand blocks of ASA

	Reference	AKI knockout	AKII knockout	AKI, AKII
				knockout
${R^{{J}_{5}}_{\textit {ASA}}}$	−1.04	−0.95	−0.66	−0.46
${R^{{J}_{8}}_{\textit {ASA}}}$	0.52	0.53	0.59	0.61
${R^{{J}_{16}}_{\textit {ASA}}}$	0.14	0.14	0.14	0.15
|*R* *ASA* *J*_5_/*R* *ASA* *J*_8_ |	2.01	1.80	1.13	0.76
|*R* *ASA* *J*_5_/*R* *ASA* *J*_16_ |	7.56	6.85	4.60	3.17

Total response coefficients of *J*_5_, *J*_8_ and *J*_16_ towards ASA at the steady-state and the distribution of flux control between the supply and demand blocks according to the principle illustrated in Eq. 6 are presented. Control analysis was performed for the reference model and each of the knockouts with the concentration of the linking metabolite between supply and demand fixed at the steady-state value in each case (see Table S2 in Additional file 6)

## Discussion

In this paper we set out to investigate multiple routes of interaction between reaction blocks in metabolic systems using the framework of generalised supply-demand analysis. This allowed us to identify routes of regulation and quantify the contributions of various reaction blocks towards metabolic behaviours such as flux responses. The two models investigated were chosen on the basis of the mechanism whereby multiple regulatory routes are mediated: moiety-conserved cycles connect different metabolic branches in the pyruvate branch model, while allosteric inhibition of various isoenzymes allows for communication between separate ends of the pathway in the aspartate model. In both cases, our analysis provided insight into how different routes of interaction contribute to the overall behaviour of the systems.

In the model of pyruvate metabolism in *Lactococcus lactis* multiple routes of interaction between reaction blocks are brought about by the moiety-conserved cycles of ATP/ADP, NADH/NAD ^+^ and acetyl-CoA/CoA. The regulation of this pathway in *L. lactis* is relatively well understood due to the extensive study of this industrially significant organism [20]. Moreover, a number of newer and more extensive models for *L. lactis* central carbon metabolism exist [21–24], but most only include a simplified pyruvate branch metabolism. The most recently published model [24], for instance, lumps certain reactions such as acetaldehyde dehydrogenase and alcohol dehydrogenase, and phosphotransacetylase and acetate kinase together. Since our primary aim was to delineate and quantify regulatory routes of interaction, we chose to investigate the original model by Hoefnagel et al. [11], because it incorporates in the most detail the multiple interactions between the different branches of pyruvate metabolism due to the presence of moiety-conserved cycles. While an extended version of this model exists [21], which also incorporates glycolytic reactions in detail, the simpler version was chosen, as both have identical representations of the pyruvate branch metabolism and the extended model has not been published in full detail. We opted for an exploratory approach, focusing on the application of GSDA to this model in order to extract information about the effects of ATP/ADP and NADH/NAD ^+^ on various reaction blocks via multiple routes. This generated results that matched previous observations, but allowed us to offer a novel quantitative explanation.

The most striking result was that moiety ratios far from the steady-state caused unexpected flux responses in two reaction blocks: The ATP/ADP-producing flux *J*_5_ responded positively to low ATP/ADP-values, while the NADH/NAD ^+^-consuming flux *J*_6_ responded negatively to high NADH/NAD ^+^-values. The reactions, respectively catalysing the last and first reactions in these reaction blocks, are not product activated or substrate inhibited in the model [11], therefore the observed flux responses had to originate from multiple routes of regulation. This was confirmed by utilising partial response coefficients to quantify the relative contribution of each route of interaction towards the total response of the two reaction blocks. The dominant route of regulation of its supply flux *J*_5_ by all ATP/ADP-values below 30 was via the upstream lumped glycolysis reaction (*v*_1_), rather than the direct route via the ATP-producing enzyme acetate kinase (*v*_5_). This included both the steady-state value of ATP/ADP and the ATP/ADP-range where *J*_5_ had a positive response. This is most probably an incomplete picture of regulation by ATP/ADP, as the inhibition by ATP and ADP of *L. lactis* enzymes such as lactate dehydrogenase, alcohol dehydrogenase and glyceraldehyde 3-phosphate dehydrogenase [25, 26] was not included in the model. It is conceivable that these additional routes of interaction could significantly affect the flux responses investigated here. It is, however, premature to speculate on any specifics without performing further work, due to the added complexity accompanying these interactions. Nevertheless, these results illustrate how a few routes of interaction can bring about unintuitive, non-monotonic flux responses, and how the different routes can be quantified in terms of their contributions towards these responses.

We found that while the direct route of interaction of NADH/NAD ^+^ with reaction block 6 via acetaldehyde dehydrogenase (*v*_6_) mostly determined the behaviour of *J*_6_ at steady-state, the interaction via pyruvate dehydrogenase (*v*_3_) dominated at higher NADH/NAD ^+^-values, thereby causing a decrease in *J*_6_. In spite of the limitations of this model, this corresponds well with the previously established role of redox balance in regulating pyruvate flux distribution, where low NADH/NAD ^+^-values are associated with mixed-acid fermentation and higher values with homolactic fermentation [27–30]. While a high sensitivity of lactate dehydrogenase towards NADH/NAD ^+^ [27] was not observed here, the reduction of flux towards acetyl-CoA (*J*_3_) by inhibition of pyruvate dehydrogenase, and therefore also the ethanol flux (*J*_6_), in response to the increase in NADH/NAD ^+^ was indeed observed [27–30]. Due to the structure of this pathway, one may conclude that reduced *J*_3_ should lead to a reduction in *J*_6_, but in reality matters are not that simple. While *J*_6_ and *J*_3_ did decrease concomitantly at higher NADH/NAD ^+^-values, there were also values for which *J*_6_ increased while *J*_3_ decreased. The observed *J*_6_-response towards NADH/NAD ^+^ was shown to be a combination of complementary and competing non-monotonic effects that varied in importance with the value of the moiety ratio, thereby highlighting the utility of a model analysis tool such as GSDA for providing quantitative explanations for observed system behaviour.

Unsurprisingly, NADH/NAD ^+^ has been shown to determine pyruvate flux distribution in other organisms, such as *Saccharomyces cerevisiae* [31] and *Escherichia coli* [32, 33], in a similar manner to *L. lactis* [28]. For these organisms, similar analyses could improve our understanding not only of their individual metabolisms, but also of pyruvate distribution in general. Furthermore, in addition to the role of NADH/NAD ^+^ in energy metabolism, NAD ^+^ and NADP ^+^ also play roles ranging from antioxidation to telomere metabolism as discussed in a comprehensive review by Ying [34]. While the approach used here may not be appropriate for the study of every role of NADH/NAD ^+^, its application could shed light on the specific regulatory role of NADH/NAD ^+^ in other pathways.

The second model investigated describes aspartate-derived amino acid synthesis in *Arabidopsis thaliana*. Here we focussed on the regulation of the ASA supply block by ASA itself. This reaction block was of special interest as its first step is catalysed by four AK isoenzymes, two of which are inhibited by Thr, and the other two inhibited by Lys. Each of these inhibitors is produced by a separate metabolic branch, with two isoenzymes catalysing the initial step of each ASA-consuming branch. These features enable ASA to communicate with its supply block via multiple routes.

Our results show that the majority of regulation of the ASA supply block did not occur via the interaction of ASA with its producing reaction, but rather by interaction with its demand blocks, which in turn affected the concentrations of the AK inhibitors Lys and Thr. One intuitively expects that regulation should occur via the shortest route, especially when taking into account the relatively high sensitivity of aspartate-semialdehyde dehydrogenase towards ASA at the steady-state in this system. The most unexpected result is the apparent importance of AKI and AKII in the ASA supply block regulation, in spite of their low contribution towards total flux. Previously, Curien et al. [19] analysed knockout simulations of this model and showed that AKI and AKII could compensate for the loss of AK1 and AK2 in terms of flux, thereby providing redundancy and confirming the idea that the role of isoenzymes is to provide robustness to the system [35, 36]. We, however, postulate that these reactions play an additional regulatory role which is largely decoupled from their function as carriers of flux, and that AKI and AKII provide robustness in terms of this role for each other. Our own knockout simulations of AKI and AKII showed that, while total flux remained largely unchanged for both knockouts (showing these reactions to be practically redundant in terms of flux), they were not redundant in terms of regulatory importance. While the loss of AKI could be compensated for by AKII, the reverse was not true, and a shift of regulation towards the Lys branch took place. However, in spite of the inadequacy of AKI as a substitute for AKII in terms of regulatory function and flux contribution, it was much more effective in emulating the former function than the latter. Furthermore, a double knockout of AKI and AKII decreased the total regulation of *J*_5_ by ASA to less than 50 % of the wild-type, shifting flux control from the demand block to, less optimally [13], the supply block. This means that increases or decreases in Thr demand will no longer lead to effective regulation of the ASA flux.

The source of the flux responses of both models was investigated by separating the partial response coefficients into their control and elasticity components. In this way the flux response coefficients could be classified as originating predominantly from a local (i.e. enzyme) property or from a system property. We broadly classified a control or elasticity coefficient as having a dominant contribution towards the response coefficient in two different ways: either (1) the magnitude of one coefficient outweighs the contribution of the other, or (2) one coefficient changes in value over a parameter range while the other remains relatively constant; the varying coefficient therefore determines the change in response coefficient. In certain cases we found that the elasticity coefficients dominated the flux response (e.g., the large values of $\phantom {\dot {i}\!}{{~\!\!}^{v_{7}}R^{J_{6}}_{\phi _{N}}}$ and $\phantom {\dot {i}\!}{{~\!\!}^{v_{6}}R^{J_{6}}_{\phi _{N}}}$ at *ϕ*_*N*_=0.0012 due to the huge values of $\phantom {\dot {i}\!}{\varepsilon ^{v_{7}}_{\phi _{N}}}$ and $\phantom {\dot {i}\!}{\varepsilon ^{v_{6}}_{\phi _{N}}}$ in the pyruvate model), while in other cases control coefficients dominated (e.g., the low value of $\phantom {\dot {i}\!}{{~\!\!}^{v_{5}}R^{J_{5}}_{\textit {ASA}}}$ at the steady-state due to the low value of $\phantom {\dot {i}\!}{C^{J_{5}}_{v_{5}}}$ in the aspartate model).

The work presented here reiterates the fact that metabolic systems can exhibit complex behaviour that cannot be predicted from simple inspection of the reaction network. Even when the network structure is considered together with enzyme-kinetic properties, in some cases understanding does not emerge intuitively. Furthermore, as the size and complexity of a system increases, so too does the variety of possible behaviours. Examples are the instances of apparent substrate inhibition and product activation in the pyruvate model (Figs. 4a and 6a), where no such mechanisms exist on the enzyme level. Another example is in the aspartate model, where the seemingly predictable negative response of *J*_5_ towards ASA is not due to product inhibition of ASADH, but rather due to upstream inhibition of the aspartate kinase isoenzymes by inhibitors downstream from ASA (Fig. 9). The phenomena in both these cases stem from the existence of multiple routes of interaction between metabolites and reactions and were only brought to light through simulation and analysis. We could not only demonstrate unintuitive behaviour, but also quantify the contribution of the different routes of interaction towards bringing about this behaviour.

It is possible to analyse regulation at a deeper level by analysing the control coefficients that form part of the partial response coefficients in term of so-called control patterns [37]. A control pattern can be understood as a ‘chain of local effects’ that propagates through a metabolic pathway following a perturbation in a pathway parameter such as enzyme concentration. Each control pattern is a scaled product of elasticity coefficients, and each control coefficient is a sum of control patterns. Going even deeper, it is possible to partition the constituent elasticity coefficients into additive kinetic and thermodynamic terms [38]. The fact that control coefficients are complex functions of elasticity coefficients is also the reason why certain control coefficients, such as ${C^{J_{6}}_{v_{3}}}$ and ${C^{J_{6}}_{v_{6}}}$ in the pyruvate model, responded non-monotonically to changing parameters, whereas the elasticities themselves responded monotonically. In this study there was only one situation where we could unambiguously relate an observed flux response to one of the terms in an elasticity coefficient: in the pyruvate model the infinite elasticities of reactions 6 and 7 towards *ϕ*_*N*_ at *ϕ*_*N*_=0.0012 were due to these reactions being near equilibrium, a situation where for any reaction the thermodynamic term determines the value of its substrate and product elasticity coefficients. This observation was therefore only possible due to infinite elasticity coefficients being a very obvious and well-known sign of a reaction near equilibrium. To fully understand the pathways investigated here in terms of control patterns or in terms of the thermodynamics and kinetics of the pathway enzymes will require further analysis.

Both pathways studied here have potential biotechnological and industrial applications. *L. lactis* is an important organism in the dairy industry where the desirable products of pyruvate metabolism, such as diacetyl and acetaldehyde, are not always produced in equally desirable quantities [11, 27, 29]. Modification of *L. lactis* to increase these products is therefore an appealing prospect. While *A. thaliana* itself is not industrially important, it is used as a model organism for plant species in general. Here the modification of the aspartate-derived amino acid synthesis pathway to increase the production of the essential amino acids threonine and lysine could lead to the development of crops with increased nutritional value [39, 40]. However, the development of rational metabolic engineering strategies to leverage the metabolisms of these organisms requires a detailed understanding of their function. Application of the methods demonstrated in this paper can act as a stepping stone towards the development of such strategies by providing additional insights into mechanisms of metabolic regulation.

## Conclusions

The regulation of the supply and demand blocks of a specific intermediate *by the intermediate itself* becomes convoluted when these reaction blocks can also interact through other intermediates, and not only through the linking intermediate. Generalised supply-demand analysis is a framework that allows for the identification of regulatory features of a metabolic pathway, one of which is the quantitative relative contribution of multiple routes of regulation of supply or demand blocks by the intermediate that links them.

Here we have demonstrated the use of generalised supply-demand analysis in disentangling various routes of regulation in a model of pyruvate metabolism where the involvement of the conserved moieties ATP/ADP and NADH/NAD ^+^ in multiple reactions caused counter-intuitive responses in the fluxes of their producing and consuming blocks, and a model of aspartate metabolism where aspartate-semialdehyde could communicate with its supply block via multiple branching routes that were enabled by allosteric affectors and isoenzymes. Our findings showed that indirect routes of interaction between an intermediate and a reaction block can play a more significant role than the direct route.

We also demonstrated the utility of using a variety of analytic techniques in the further analysis of metabolic models. Both models provided novel results in spite of their having been studied by their original authors in the past [11, 12]. Further analysis with complementary tools such as control-pattern analysis would allow us to shed light on the source of the observed metabolic control in terms of chains of local effects [37, 41] and enzyme sensitivities in terms of thermodynamic and kinetic contributions [38]; computational implementations of these tools are currently in development.

## Availability of supporting data

The original SBML versions of the models used in this paper can be found online in the BioModels Database [4] under the unique BioModels IDs BIOMD0000000017 and BIOMD0000000212 for the pyruvate branch and the aspartate-derived amino acid synthesis pathways, respectively. The PySCeS MDL and SBML versions of these models, together with a script to recreate the results presented here, are attached as “Additional files” (see below). PySCeS MDL files of the models were obtained as described under “Methods”.

## Additional files

Additional file 1
**PySCeS model descriptor language file for the pyruvate model, as required by the IPython notebook provided as Additional file** 5 **(see instructions within notebook).** (PSC 2 kb)

Additional file 2
**PySCeS model descriptor language file for the aspartate model, as required by the IPython notebook provided as Additional file** 5 **(see instructions within notebook).** Software needed for Additional files 1 and 2: PySCeS (see http://pysces.sourceforge.net/docs/userguide.html). (PSC 2 kb)

Additional file 3
**SBML [** 18 **] version of the pyruvate model.** (XML 40 kb)

Additional file 4 JWSOnline (http://jjj.biochem.sun.ac.za/) Copasi (http://www.copasi.org) Other SBML-compliant simulators (http://sbml.org/SBML_Software_Guide/SBML_Software_Matrix) may also work. (XML 31 kb)

Additional file 5
**An IPython notebook file in which the results shown in Figs.** 4, 5, 6, 7, 8, 9, 10, 11, 12 **and** 13,** Table** 3 **and Additional file** 6 **are generated using PySCeS and PySCeSToolbox.** This notebook requires the model files provided in Additional files 1 and 2. Software needed: IPython notebook with PySCeS and PySCeSToolbox as requirements (see https://github.com/PySCeS/PyscesToolbox for full requirements and installation instructions). (IPYNB 5 kb)

Additional file 6
**A pdf document containing Table S1 (metabolic control analysis) and Table S2 (steady-state analysis) for the aspartate metabolism model.** (PDF 75 kb)

## Abbreviations

GSDA: Generalised supply-demand analysis
MCA: Metabolic control analysis
PySCeS: The Python simulator for cellular systems
Ac: Acetate
Acal: Acetaldehyde
Acet: Acetoin
Aclac: Acetolactate
Acp: Acetyl phosphate
Glc: Glucose
Lac: Lactate
But: 2,3-Butanediol
Pyr: Pyruvate
EtOH: Ethanol
Ado-Met: S-adenosylmethionine
ASA: Aspartate-semialdehyde
Asp: Aspartate
AspP: Aspartyl phosphate
Cys: Cysteine
Hser: Homoserine
Ile: Isoleucine
Lys: Lysine
PHser: Phosphohomoserine
Thr: Threonine
Val: Valine
AK: Aspartate kinase
ASADH: Aspartate-semialdehyde dehydrogenase
HSDH: Homoserine dehydrogenase
DHDPS: Dihydrodipicolinate synthase
