# Supplementary material for: Tracing regulatory routes in metabolism using generalised supply-demand analysis
Source: BMC Syst Biol. 2015 Dec 3;9:89. doi: 10.1186/s12918-015-0236-1 (PMC4669674; doi:10.1186/s12918-015-0236-1)
Supplement: Additional file 6 — A pdf document containing Table S1 (metabolic control analysis) and Table S2 (steady-state analysis) for the aspartate metabolism model. (PDF 75 kb) [file 12918_2015_236_MOESM6_ESM.pdf]

Tracing regulatory routes in metabolism using generalised supply-demand analysis

C.D. Christensen, J.-H.S. Hofmeyr & J.M. Rohwer

Additional File 1 — Additional Tables

Table S1: Metabolic control analysis of J_5, J_9, J_{14} and J_{15} for the aspartate metabolism model. Partial response coefficients and their associated elasticity and control coefficients are shown. Control analysis was performed with the concentration of linking metabolite between supply and demand fixed at the reference steady-state value in each case (see Table 2 in main text).

Elasticity coefficient		Control coefficient		Partial response coefficient	
$\varepsilon^{v_5}_{ASA}$	-2.517	$C^{J_5}_{v_5}$	0.029	${}^{v_5}R^{J_5}_{ASA}$	-0.072
$\varepsilon^{v_6}_{ASA}$	1.004	$C^{J_5}_{v_6}$	-0.275	${}^{v_6}R^{J_5}_{ASA}$	-0.276
$\varepsilon^{v_7}_{ASA}$	1.001	$C^{J_5}_{v_7}$	-0.320	${}^{v_7}R^{J_5}_{ASA}$	-0.320
$\varepsilon^{v_{15}}_{ASA}$	1.002	$C^{J_5}_{v_{15}}$	-0.337	${}^{v_{15}}R^{J_5}_{ASA}$	-0.338
$\varepsilon^{v_{14}}_{ASA}$	1.000	$C^{J_5}_{v_{14}}$	-0.037	${}^{v_{14}}R^{J_5}_{ASA}$	-0.037
$\varepsilon^{v_1}_{Thr}$	-2.357	$C^{J_9}_{v_1}$	0.015	${}^{v_1}R^{J_9}_{Thr}$	-0.035
$\varepsilon^{v_2}_{Thr}$	-1.763	$C^{J_9}_{v_2}$	0.078	${}^{v_2}R^{J_9}_{Thr}$	-0.137
$\varepsilon^{v_6}_{Thr}$	-0.327	$C^{J_9}_{v_6}$	0.193	${}^{v_6}R^{J_9}_{Thr}$	-0.063
$\varepsilon^{v_7}_{Thr}$	-0.025	$C^{J_9}_{v_7}$	0.224	${}^{v_7}R^{J_9}_{Thr}$	-0.006
$\varepsilon^{v_3}_{Lys}$	-0.857	$C^{J_{14}}_{v_3}$	0.113	${}^{v_3}R^{J_{14}}_{Lys}$	-0.097
$\varepsilon^{v_4}_{Lys}$	-0.833	$C^{J_{14}}_{v_4}$	0.534	${}^{v_4}R^{J_{14}}_{Lys}$	-0.445
$\varepsilon^{v_{15}}_{Lys}$	-1.630	$C^{J_{14}}_{v_{15}}$	-0.210	${}^{v_{15}}R^{J_{14}}_{Lys}$	0.342
$\varepsilon^{v_{14}}_{Lys}$	-1.956	$C^{J_{14}}_{v_{14}}$	0.977	${}^{v_{14}}R^{J_{14}}_{Lys}$	-1.911
$\varepsilon^{v_3}_{Lys}$	-0.857	$C^{J_{15}}_{v_3}$	0.113	${}^{v_3}R^{J_{15}}_{Lys}$	-0.097
$\varepsilon^{v_4}_{Lys}$	-0.833	$C^{J_{15}}_{v_4}$	0.534	${}^{v_4}R^{J_{15}}_{Lys}$	-0.445
$\varepsilon^{v_{15}}_{Lys}$	-1.630	$C^{J_{15}}_{v_{15}}$	0.790	${}^{v_{15}}R^{J_{15}}_{Lys}$	-1.288
$\varepsilon^{v_{14}}_{Lys}$	-1.956	$C^{J_{15}}_{v_{14}}$	-0.023	${}^{v_{14}}R^{J_{15}}_{Lys}$	0.045

Table S2: Steady-state concentrations and fluxes for aspartate metabolism in the reference model and for knockouts. Rates are in $\mu M\ s^{-1}$ and concentrations are in μM).

	Reference	AKI knockout	AKII knockout	AKI,AKII knockout
ASA	0.96	0.95	0.88	0.84
Lys	69.16	68.69	65.99	64.74
Thr	296.93	285.74	228.99	206.81
J_1	0.018	-	0.033	-
J_2	0.095	0.102	-	-
J_3	0.158	0.159	0.165	0.169
J_4	0.745	0.750	0.780	0.794
J_5	1.016	1.011	0.978	0.962
J_6	0.324	0.323	0.320	0.317
J_7	0.377	0.372	0.346	0.334
J_{14}	0.031	0.031	0.031	0.031
J_{15}	0.284	0.284	0.280	0.279
